# Cardioprotective Effects of Ferulic Acid Through Inhibition of Advanced Glycation End Products in Diabetic Rats with Isoproterenol-Induced Myocardial Infarction

**DOI:** 10.3390/ph18030319

**Published:** 2025-02-25

**Authors:** Sarah Ouda Bekheit, Eman Kolieb, El-Sayed E. El-Awady, Maha Abdullah Alwaili, Afaf Alharthi, Dina M. Khodeer

**Affiliations:** 1 Department of Pharmacology and Toxicology, Faculty of Pharmacy, Suez Canal University, Ismailia 41522, Egypt; saraouda97@gmail.com (S.O.B.); sayed.alawadi@pharm.suez.edu.eg (E.-S.E.E.-A.); 2 Department of Medical Physiology, Faculty of Medicine, Suez Canal University, Ismailia 41522, Egypt; eman.kolieb@med.suez.edu.eg; 3 Department of Biology, College of Science, Princess Nourah bint Abdulrahman University, P.O. Box 84428, Riyadh 11671, Saudi Arabia; maalwaele@pnu.edu.sa; 4 Department of Clinical Laboratory Sciences, College of Applied Medical Sciences, Taif University, P.O. Box 11099, Taif 21944, Saudi Arabia; a.awwadh@tu.edu.sa

**Keywords:** advanced glycation end products and their receptors, diabetes mellitus, ferulic acid, isoproterenol, myocardial infarction, oxidative stress

## Abstract

Background/Objectives: Myocardial infarction (MI) and diabetes pose significant health challenges globally, necessitating the development of innovative medication strategies to improve outcomes in affected populations. This research aimed to determine the defensive impact of ferulic acid (FA) against isoproterenol-induced myocardial infarction (MI) in diabetic rats. Methods: A group of male rats was partitioned into five distinct groups: control group, diabetic group, diabetic + MI, diabetic + MI + 20 mg/kg FA, and diabetic + MI + 40 mg/kg FA. The experimental groups received isoproterenol (ISO) subcutaneously at a dosage of 50 mg/kg body weight for two consecutive days. Results: The outcome was severe cardiac toxicity, as shown by changes in electrocardiogram (ECG) rhythm and a substantial increase in blood cardiac enzymes such as creatinine kinase (CK-MB), cardiac troponin I (cTnI), and lactate dehydrogenase (LDH). Additionally, there was a surge in inflammatory cytokines, like tumor necrosis factor-alpha (TNF-α), and a disruption of the antioxidant system, evidenced by a rise in malondialdehyde (MDA) content. Moreover, there was a rise in cardiac receptor of advanced glycation end products (RAGE). Treatment with FA with escalating dosages of 20 and 40 mg/kg b.w. effectively mitigated changes in serum cardiac enzymes and improved the cellular architecture, which was evaluated by histopathological examination. Conclusions: In conclusion, in a dose-dependent manner, FA successfully showed a cardioprotective effect against ISO-induced cardiac toxicity in diabetic rats, as shown by the improvement in ECG findings, normalization of serum cardiac biomarkers, and augmentation of the endogenous antioxidant system. Therefore, the aforementioned data indicate that ferulic acid may potentially have a protective effect on MI patients who have diabetes mellitus.

## 1. Introduction

Diabetes is a metabolic disease marked by prolonged high glucose levels in the bloodstream caused by insufficient insulin production or compromised insulin function, which can be attributed to poor pancreatic β-cell activity or insulin resistance. The two pathophysiologies are distinctive features of type 2 diabetes (T2D), the most prevalent form of diabetes [1]. This condition is a metabolic disease that disrupts the metabolic processes of large biological molecules [2]. Diabetes-related cardiovascular disease was the principal reason for the death of 17.9 million people globally in 2016, which affected 31% of all fatalities worldwide [3]. Moreover, the prevalence of CVDs, including stroke and myocardial infarction (MI), surged from 257 million cases in 1990 to 550 million cases in 2019 [4,5,6]. The pathophysiology of MI is complex and involves a combination of various mechanisms, for example, oxidative stress, contractile dysfunction, endothelial damage, and cell death by apoptosis or necrosis [7]. Patients with type 2 diabetes have a two to four times greater risk of CVD mortality than non-diabetic patients [8]. Myocardial infarction (MI) and diabetes are major contributors to global morbidity and mortality, presenting substantial challenges to healthcare systems worldwide [9]. These conditions frequently coexist, complicating patient management and prognosis. The interplay between diabetes and MI involves complex pathophysiological mechanisms, including impaired glucose metabolism, chronic inflammation, and increased thrombotic activity, which accelerate the progression of atherosclerosis and heighten the risk of cardiac events [10]. Current therapeutic approaches often fall short in fully addressing the needs of this patient demographic, highlighting an urgent need for innovative medication strategies. By focusing on the development of treatments that target specific pathological processes at the intersection of MI and diabetes, researchers aim to significantly improve clinical outcomes and enhance the quality of life for these patients [11].

Oxidative stress is the pivotal mediator in causing damage to the heart muscle secondary to ischemia and reperfusion in type 2 diabetes, distorting the diabetic myocardium structure and function [12]. The condition may be caused directly or indirectly by elevated blood sugar levels, high fats in the blood, and decreased insulin sensitivity. Reactive oxygen species (ROS) are highly reactive molecules resulting from their ability to accept electrons. In their higher concentrations, ROS cause many degenerative diseases. Several pharmaceutical compounds can generate ROS and induce oxidative stress [13]. For instance, isoproterenol (ISO) is known to be one of the agents that generate extremely cytotoxic ROS [14]. Moreover, isoproterenol stimulates the secretion of inflammatory cytokines, including TNF-α, IL-1β, and IL-6 [15].

Advanced glycation end products (AGEs) are potent oxidizing substances produced by a nonenzymatic glycation process involving nucleic acids, free amino acids, reducing sugars, and lipids. Multiple endogenous and exogenous sources can produce AGEs [16]. The production of AGEs is irreversible and generally slow in physiological conditions. In contrast, many factors may accelerate the formation of AGEs, such as oxidative stress, hypoxia, hyperglycemia, insulin resistance, obesity, and aging [17]. AGEs play a crucial role in triggering vascular complications of diabetes by inducing oxidative stress, inflammation, and thrombogenic and fibrotic reactions [18]. The next approach to preventing or postponing vascular problems in people with diabetes involves inhibiting the synthesis of AGEs. OPB-9195 and aminoguanidine, which are synthetic medications, can hinder the creation of AGEs. However, it is important to note that these drugs have a very high level of toxicity [19]. As a result, several studies have focused on natural products as sources of potential inhibitors of AGE development, prioritizing their high safety and low toxicity. Phenolic acids and flavonoids derived from plants have recently been shown to be effective glycation inhibitors [20].

Ferulic acid (FA), also known as 4-hydroxy-3-methoxy cinnamic acid (C_10_H_10_O_4_), is a phenolic compound found in various plants and foods of plant origin. Some examples are tomatoes [21], sweet corn [22], and wheat [23]. FA is a bioactive compound with various functions, including antidiabetic, anti-inflammatory, and antioxidant functions. It can eliminate excessive ROS, function as a powerful scavenger of free radicals, such as hydroxyl and peroxyl radicals, and prevent lipid peroxidation [24]. Additionally, FA can inhibit platelet aggregation, reduce thrombosis [25], and prevent liver cholesterol synthesis by lowering blood lipid concentrations. Consequently, it can prevent coronary heart disease [26]. Ferulic acid, a natural phenolic compound found in various plants and foods, is beneficial in managing diabetes and cardiovascular diseases. Ferulic acid helps improve insulin sensitivity and reduce blood sugar levels by combating oxidative stress and inflammation, which are common complications in diabetic patients [27]. Ferulic acid exhibits strong antioxidant properties for cardiovascular diseases that can protect against heart tissue damage by reducing oxidative stress and inflammation. Additionally, it has been shown to help regulate blood pressure and cholesterol levels, further supporting heart health and preventing the progression of cardiovascular diseases [28]. While the general inhibitory effects of FA on AGEs have been reported in previous studies, our study provides new insights into the specific application of FA in a clinically relevant model of diabetic MI. By exploring FA’s effects on AGE–RAGE-mediated oxidative stress and inflammation in this complex pathological setting, we aim to contribute to the growing body of evidence supporting FA’s therapeutic potential in diabetic cardiovascular complications. Furthermore, this study assesses whether ferulic acid has a cardioprotective effect by improving ECG findings, normalizing serum cardiac biomarkers, and augmenting the endogenous antioxidant system. Consequently, these results may spotlight the benefits of natural products in preventing the complications of long-term disorders like diabetes.

## 2. Results

### 2.1. The Effect of Ferulic Acid on Body Weight, Left Ventricle Weight (LV), and Heart Weight (HtWt)-to-Body Weight (BWt) Ratios

Table 1 shows no significant difference between groups for body weight at baseline. At the same time, the end findings of the investigation revealed a substantial variation in body weight across the groups. The highest mean changes in body weight were recorded in the diabetic + MI group (34.27%), followed by the diabetic group (29.9%) and diabetic + MI + FA (20 mg/kg) group (22.0%), while the lowest mean was recorded in the diabetic + MI + FA (40 mg/kg) group (17.9%), followed by the control group (18.0%). Regarding the HtWt and LV weight ratios, the highest mean ratio was recorded in the diabetic + MI group, followed by the diabetic and diabetic + MI + FA (20 mg/kg) groups, while the lowest was recorded in the control group. Statistical analysis showed significant differences between groups for these variables.

The percentage change in body weight (BWt) was determined using the following formula: % change BWt = [(final BWt − baseline BWt)/baseline BWt] × 100. The initial body weight (BWt) was measured at the beginning of the trial, whereas the final BWt was measured after week 10. The data are shown as the mean value plus or minus the standard deviation (mean ± SD). They were analyzed using a one-way analysis of variance (ANOVA) followed by Bonferroni’s post hoc test, with the significance level set at *p* ˂ 0.05, n = 8. Significant differences were observed between the control group and all diabetic groups (*p* < 0.05). The diabetic + MI + FA 40 mg/kg group showed significant improvements compared to the diabetic + MI group (*p* < 0.05).

The effect of ferulic acid on fasting blood glucose:

Statistical analysis revealed significant differences between BGL, insulin, and HOMA-IR among the groups using one-way ANOVA at *p* < 0.05. For BGL, the highest mean values were recorded in the diabetic + MI group (10.60 ± 2.04), followed by the diabetic group (7.90 ± 0.52), diabetic + MI + FA (20 mg/kg) group (6.1 ± 0.10), and diabetic + MI + FA (40 mg/kg) group (6.0 ± 0.26), while the lowest value was recorded in the control group (5.13 ± 0.06). Regarding insulin, the highest mean values were recorded in the diabetic + MI group (37.20 ± 2.65), followed by the diabetic + MI + FA (20 mg/kg) group (20.60 ± 2.56), diabetic group (17.20 ± 0.20), and diabetic + MI + FA (40 mg/kg) group (9.47 ± 0.95), while the lowest value was recorded in the control group (4.90 ± 0.26). For HOMA-IR, the same trend as for insulin was found, where the highest mean values were recorded in the diabetic + MI group (17.60 ± 3.99), followed by the diabetic + MI + FA (20 mg/kg) group (6.43 ± 0.91), diabetic + MI + FA (20 mg/kg) group (5.70 ± 0.44), and diabetic + MI + FA (40 mg/kg) group (2.97 ± 0.60), while the lowest value was recorded in the control group (1.77 ± 0.06) (Table 2).

Data are presented as the mean ± SD and analyzed using a one-way analysis of variance (ANOVA) followed by Bonferroni’s post hoc test at a significance level of *p* ˂ 0.05, n = 8. The diabetic + MI group had significantly higher glucose levels compared to the control group (*p* < 0.001). FA treatment at 40 mg/kg significantly reduced glucose levels compared to the diabetic + MI group (*p* < 0.05).

a Compared to control group.

b Compared to diabetic group.

c Compared to diabetic + MI group.

### 2.2. The Effect of Ferulic Acid on ECG Patterns

**Figure 1:**
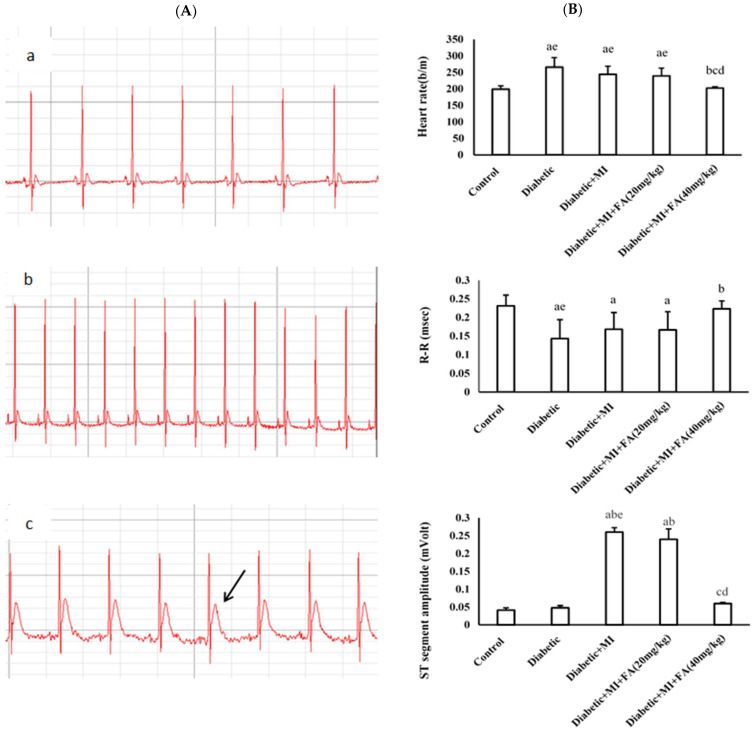

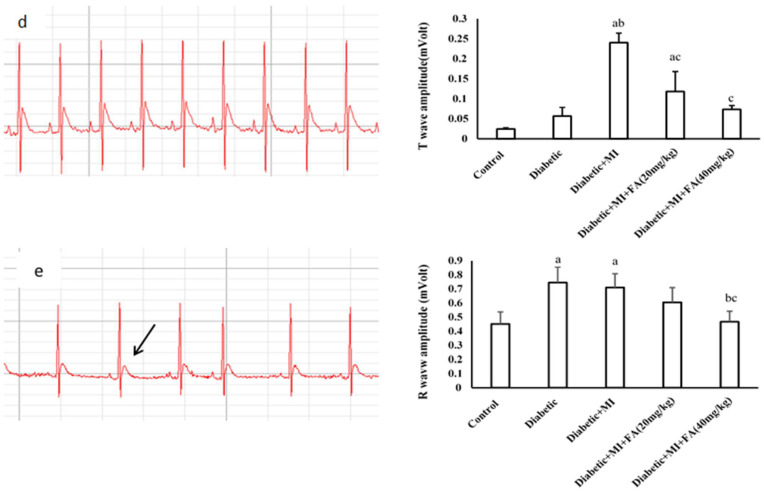
The ECG readings in the various treated groups. (**A**) The electrocardiographic impact of different FA dosages on ISO-induced MI in diabetic rats. (**B**) ECG parameters H.R., R-R interval, S.T. segment amplitude, T wave amplitude, and R wave amplitude in experimental rats pretreated with FA (20, 40 mg/kg) in an ISO-induced MI diabetic paradigm. (**a**) Control group, (**b**) diabetic group, (**c**) diabetic + MI group, (**d**) diabetic + MI + FA 20 mg/kg group, and (**e**) diabetic + MI + FA 40 mg/kg group, arrow shows reduced the T wave amplitude to a considerably greater extent than a lower dose (FA 20 mg/kg). Bars with various letters are significantly different according to Bonferroni’s post hoc test at the *p* < 0.001 level (n = 8). Data are expressed as mean ± S.D. ECG: electrocardiogram; FA: ferulic acid; ISO: isoproterenol; MI: myocardial infarction; H.R.: heart rate.

After the examination, the control group displayed a standard electrocardiogram (ECG) pattern, which included a normal heart rate (H.R.), R-R interval, S.T. segment amplitude, and T wave amplitude. Both the diabetic group and the diabetic + MI group showed a statistically significant rise in H.R., S.T. segment amplitude, and T wave amplitude and a marked reduction in the R-R interval compared to the control group. Figure 1A,B demonstrate that compared to the control group, the H.R. of the diabetic and diabetic + MI groups increased significantly (*p* < 0.001). Rats administered FA (40 mg/kg), on the other hand, had a significantly lower H.R. than the diabetic and diabetic + MI groups (*p* < 0.001). Compared to a lower dose of FA (20 mg/kg), a higher dose of FA (40 mg/kg) significantly and much more greatly lowered H.R. (*p* < 0.05).

Compared to the control group, the diabetic and diabetic + MI groups exhibited a significant reduction in the R-R interval (*p* < 0.001). When compared to the diabetic group (*p* < 0.05), the R-R interval was considerably enhanced by a larger dose of FA (40 mg/kg) compared to a lower dose of FA (20 mg/kg). This difference was also significant when compared to the diabetic + MI group. The S.T. segment amplitude of the diabetic + MI group was significantly higher than that of the control group (*p* < 0.001) and the diabetic group (*p* < 0.001). Rats administered 40 mg/kg of FA, in contrast to the diabetic + MI and FA (20 mg/kg) groups, exhibited a substantial decrease in the S.T. segment amplitude (*p* < 0.001). The T wave amplitude showed a significant increase in the diabetic + MI group compared to the control and diabetic groups (*p* < 0.001). When compared to the diabetic + MI group, a larger dose of FA (40 mg/kg) significantly reduced the T wave amplitude to a considerably greater extent than a lower dose (FA 20 mg/kg) (*p* < 0.001). The diabetic and diabetic + MI groups had a significantly larger R wave amplitude increase than the control group (*p* < 0.001). The administration of higher dosages of FA (40 mg/kg) resulted in a substantial decrease in the R wave amplitude, particularly when compared to the diabetic and diabetic + MI groups (*p* < 0.001).

### 2.3. The Effect of Ferulic Acid on Cardiac Enzymes

**Figure 2:**
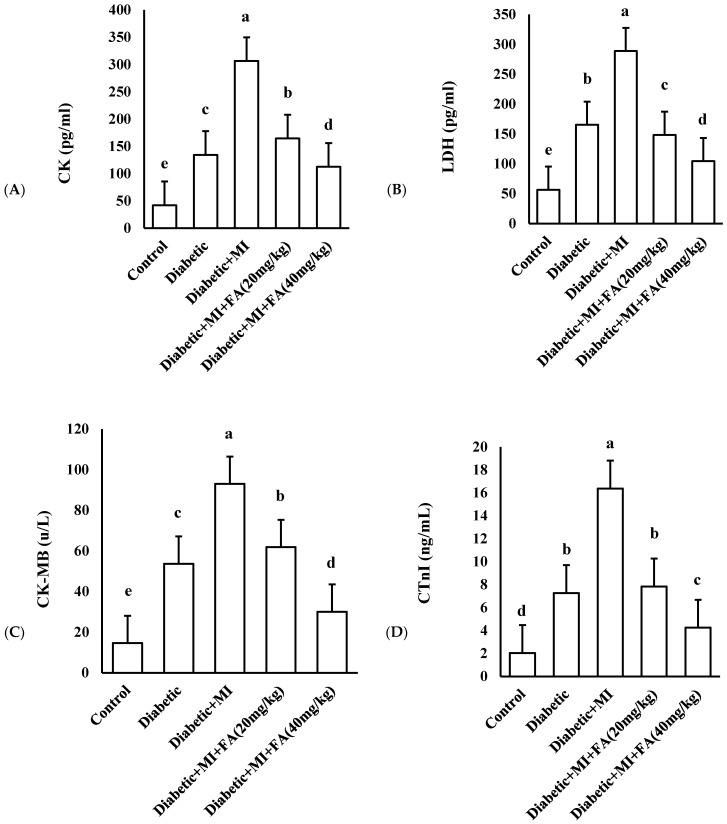
Serum levels of cardiac enzymes in the various groups. (**A**) CK, (**B**) LDH, (**C**) CK-MB, (**D**) CTnI. Data are presented as mean ± SD and analyzed using one-way ANOVA followed by Bonferroni’s post hoc test at *p* ˂ 0.05, n = 8. Bars with different letters are significantly different according to Bonferroni’s post hoc test at a significance level of *p* ˂ 0.05.

The administration of isoproterenol to diabetic rats significantly increased cardiac serum C.K., CK-MB, LDH, and cTnI levels compared to the diabetic rats that did not receive treatment and the control group that received the vehicle only. Ferulic acid administration at a dosage of 20 mg/kg did not result in noteworthy decreases in serum cardiac enzymes when compared to the group with both diabetes and myocardial infarction. However, the administration of a higher dosage of ferulic acid at 40 mg/kg led to a significant reduction (*p* < 0.05, as shown in Figure 2) in the levels of serum cardiac enzymes (C.K., CK-MB, LDH, and cTnI) when compared to the group with both diabetes and myocardial infarction.

### 2.4. Histopathological Examinations of the Cardiac Tissues in the Experimental Groups

**Figure 3:**
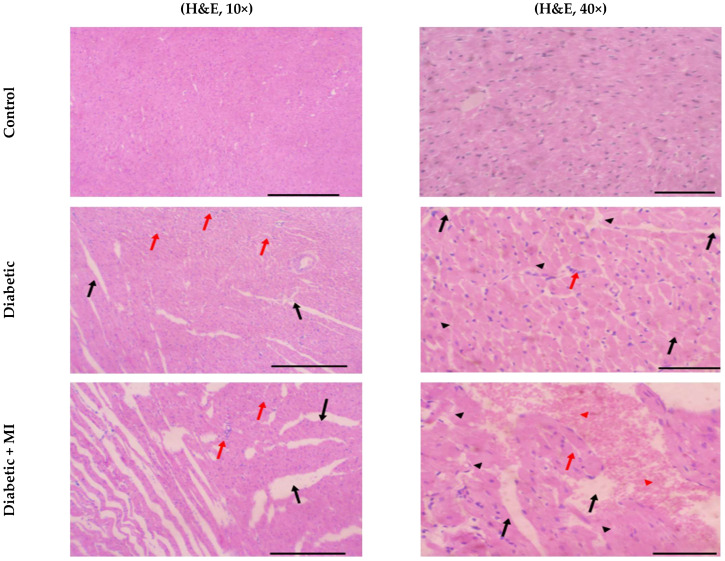

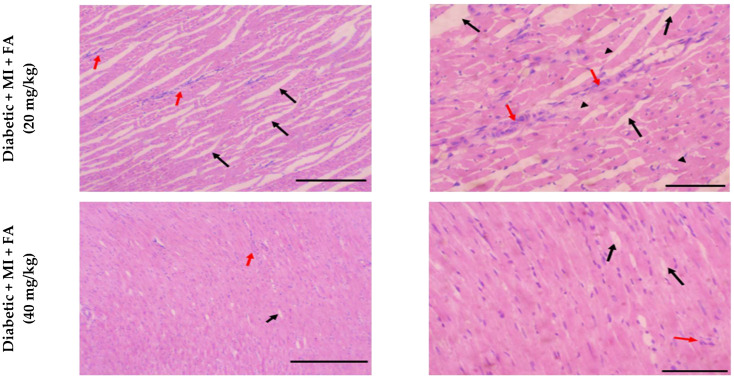
Histopathology images of hematoxylin and eosin-stained sections from the hearts of the experimental groups (×10 and ×40). MI: myocardial infarction. FA: ferulic acid. Black arrows indicate myocyte edema, and red arrows indicate mononuclear cell infiltration. Arrowhead indicate necrotic myocytes with no detected nuclei. Control group: The section from the hearts of rats treated with saline (vehicle) has a typical and healthy appearance. Diabetic group: The images indicate histological alterations of a moderate degree. Diabetic + MI group: The images indicate a rise in the quantity of neutrophils and a decline in the size and condensation of nuclei. Additionally, the tissue exhibits a loss of striations, and the sarcoplasm displays a heavily eosinophilic appearance. The diabetic + MI + FA (20 mg/kg) group exhibits histopathological changes of moderate severity. The diabetic + MI + FA (40 mg/kg) group exhibits slight histological alterations.

The histological analysis of the cardiac tissues using hematoxylin and eosin (H&E) staining demonstrated that the control group had a typical arrangement of myocardial fibers with clear striations and no noticeable signs of degenerative alterations. The evaluation of myocardial tissue in the diabetic group showed that about 75% of cardiac tissue showed evidence of injury: edema (black arrow), splitting of myofibers, and mononuclear cell infiltrate (red arrow) (H&E, 10×). Also, its higher magnification picture shows myocyte edema (black arrow), mononuclear cell infiltrate (red arrow), and necrotic myocytes (black arrowhead) (H&E, 40×). However, about 75% of cardiac tissue in the diabetic + MI group showed evidence of injury: marked edema (black arrow), splitting of myofibers, and mononuclear cell infiltrate (red arrow) (H&E, 10×). The higher magnification shows myocyte edema (black arrow), mononuclear cell infiltrate (red arrow), necrotic myocytes (black arrowhead), and areas of hemorrhage (red arrowhead) (H&E, 40×). Regarding the diabetic + MI + FA (20 mg/kg) group, there was some reduction in the extent of injury in cardiac tissue, with a slight decrease in myocyte edema (black arrow) and mononuclear cell infiltrate (red arrow) (H&E, 10×). The higher magnification shows myocyte edema (black arrow), mononuclear cell infiltrate (red arrow), and necrotic myocytes (black arrowhead) (H&E, 40×). Administration of a greater dose of FA (40 mg/kg) resulted in a significant reduction in the extent of injury in cardiac tissue, with a marked decrease in myocyte edema (black arrow) and mononuclear cell infiltrate (red arrow) (H&E, 10×). Additionally, the higher magnification shows slight myocyte edema (black arrow), mononuclear cell infiltrate (red arrow), and no necrotic myocytes (H&E, 40×) (Figure 3).

### 2.5. The Effect of Treatment with Various Doses of Ferulic Acid (20 or 40 mg/kg) on Cardiac TNF-α as an Inflammatory Marker

**Figure 4:**
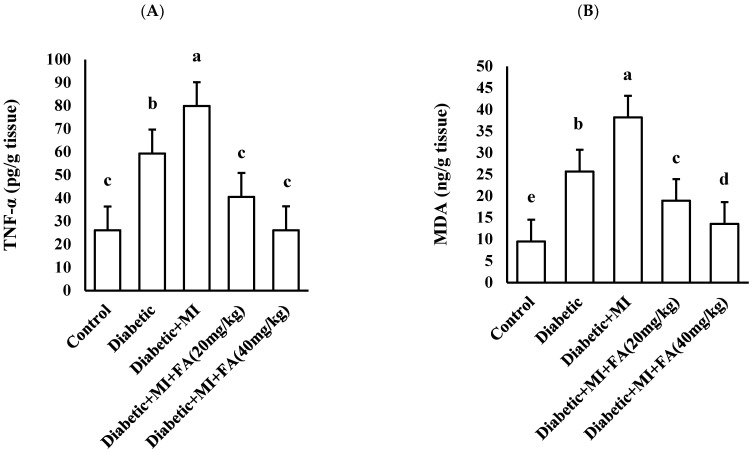
(**A**) Cardiac TNF-α and (**B**) cardiac MDA in different groups. Data are expressed as mean ± SD and analyzed using one-way ANOVA, n = 8. Bars with different letters are significantly different according to Bonferroni’s post hoc test at a significance level of *p* ˂ 0.05.

Statistical analysis showed significant differences between groups for TNF-α using one-way ANOVA at *p* < 0.05 (*p* < 0.001). The highest mean values were recorded in the diabetic + MI group (79.87 ± 4.30), followed by the diabetic group (59.33 ± 2.53) and diabetic + MI + FA (20 mg/kg) group (40.55 ± 1.44), while the control group (26.10 ± 16.80) and diabetic + MI + FA (40 mg/kg) group (26.13 ± 2.25) had the lowest values (Figure 4A).

### 2.6. The Effect of Treatment with Diverse Doses of Ferulic Acid (20 or 40 mg/kg) on Cardiac MDA as an Oxidative Marker

Statistical analysis revealed notable differences in MDA levels among the groups, as determined by one-way ANOVA with a significance level of *p* < 0.05 (*p* < 0.001). The highest mean values were recorded in the diabetic + MI group (38.17 ± 3.55), followed by the diabetic group (25.67 ± 2.35) and diabetic + MI + FA (20 mg/kg) group (18.90 ± 1.30), while the control group (9.50 ± 0.02) and diabetic + MI + FA (40 mg/kg) group (13.57 ± 1.00) had the lowest values (Figure 4B).

### 2.7. Effect of Treatment with Diverse Doses of Ferulic Acid (20 or 40 mg/kg) on Cardiac AGE–RAGE by PCR

**Figure 5:**
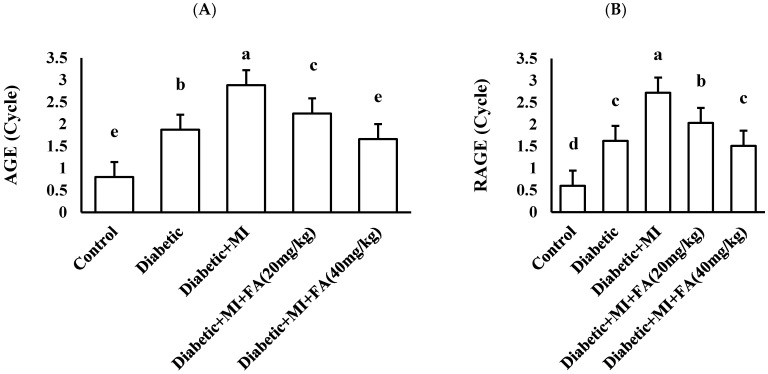
(**A**) Cardiac AGEs and (**B**) cardiac RAGE in different groups. Data are expressed as mean ± SD and analyzed using one-way ANOVA, n = 8. Bars with different letters are significantly different according to Bonferroni’s post hoc test at a significance level of *p* ˂ 0.05.

Statistical analysis indicated a significant difference among the groups for AGEs and RAGE, as assessed by one-way ANOVA at *p* < 0.05 (*p* < 0.001). For AGEs, the highest mean values were recorded in the diabetic + MI group, diabetic + MI + FA (20 mg/kg) group, diabetic group, and diabetic + MI + FA (40 mg/kg) group, with values of 2.88 ± 0.07, 2.24 ± 0.12, 1.87 ± 0.15, and 1.66 ± 0.05, respectively, while the control group had the lowest value (0.80 ± 0.02). With regard to RAGE, the highest mean values were recorded in the diabetic + MI group (2.72 ± 0.10), followed by the diabetic + MI + FA (20 mg/kg) group (2.03 ± 0.15), diabetic group (1.62 ± 0.11), and diabetic + MI + FA (40 mg/kg) group (1.51 ± 0.09), while the control group had the lowest value (0.60 ± 0.01) (Figure 5).

## 3. Discussion

The valuable outcomes of ferulic acid (FA) were evaluated against ISO-induced myocardial infarction (MI) in a diabetic rat model to determine whether these effects involve inhibiting the AGE–RAGE pathway. Induction of MI in diabetic rats resulted in various alterations in the electrocardiogram (ECG) patterns and histopathological characteristics of the heart, along with increased serum concentrations of insulin and cardiac enzymes and high levels of AGE and RAGE mRNA expression. The use of ISO to induce MI in diabetic rats is highly relevant to clinical comorbid conditions such as diabetic cardiomyopathy and stress-induced cardiac events. Diabetic patients often experience heightened sympathetic activity, which can exacerbate cardiac injury. The ISO model mimics this heightened sympathetic tone and oxidative stress, providing a valuable tool to study the effects of FA in a clinically relevant setting. Our findings demonstrate that FA mitigates ISO-induced cardiac injury, suggesting its potential to protect against stress-induced MI in diabetic patients. The present findings indicate that individuals diagnosed with type 2 diabetes face a heightened susceptibility to cardiovascular events than those without diabetes [29]. Isoproterenol (ISO) is a synthetic catecholamine molecule that is an agonist for β-adrenergic receptors. The administration of ISO through subcutaneous injection in rats results in permanent cellular damage and ultimately leads to MI [30].

Advanced glycation end products (AGEs) are a diverse group of chemicals that arise from the irreversible, nonenzymatic reactions between reducing sugars and various biomolecules, including lipids, proteins, or nucleic acids [31]. Also, AGEs can originate from external sources, such as food, or be internally produced within the body [32]. Vascular problems, particularly microvascular and cardiovascular complications, are the main causes of death and morbidity in people with diabetes [33]. AGEs can engage with their receptor (RAGE), resulting in a range of harmful consequences such as oxidative stress, apoptosis, inflammation, and the formation of what is commonly referred to as “hyperglycemia memory” [34]. The glycation and oxidative stress processes are closely interconnected, often called glycoxidation. It has been discovered that AGEs impair the activity of antioxidant systems and increase the formation of reactive oxygen species (ROS). However, it is important to highlight that some AGEs are naturally formed in oxidative conditions [35].

Based on the information presented in [36]’s model, the involvement of AGEs in different diabetic complications can be classified into two primary pathways. The first pathway involves a receptor-mediated signaling cascade, which entails the interaction between AGEs and the cell surface receptor for AGEs (RAGE). On the other hand, the second pathway involves extracellular matrix impairment caused by the cross-linking properties of AGEs.

Natural products are valuable sources of inhibitors that prevent the production of advanced glycation end products (AGEs). These inhibitors provide the benefits of being highly safe and having low toxicity levels. Recent research has demonstrated that phenolic acids and flavonoids derived from plants are potent glycation inhibitors [37]. Ferulic acid (FA) can mitigate the inflammatory response caused by AGEs by inhibiting the activation of the NF-κB and p38 MAPK signaling pathways [38]. The present findings align with previous reports indicating that FA can prevent and alleviate the progression of MI in individuals with diabetes.

Effective management of diabetes and monitoring of the glycemic index are necessary to prevent associated complications. As a result of hyperglycemia, excessive generation of free radicals occurred, subsequently inducing oxidative stress [39]. This study’s findings revealed that delivering FA resulted in a reduction in blood glucose levels and insulin levels in diabetic rats in comparison to the control group. The observed outcome revealed a dependence on the dosage administered. The data presented align with a previous report by [40], which showed that FA therapy decreased blood glucose levels. In addition, the research conducted by [41] showed that FA treatment enhanced antioxidant capacity and reduced levels of reactive oxygen species (ROS). The intervention with FA demonstrated significant enhancements in blood glucose levels and improvements in insulin secretion and sensitivity. ROS and nitrogen–oxygen species can damage cellular constituents such as proteins, DNA, unsaturated fatty acids, and cell membranes [42]. According to [43], it was observed that ROS led to an elevation in protein glycation and the formation of AGEs.

The present study observed a decrease in serum MDA levels in MI-induced diabetic rats administered FA, which aligns with prior research indicating that low doses of FA can enhance the functioning of antioxidant enzymes. This enhancement leads to the scavenging of free radicals, recognized as the primary contributors to tissue damage [39]. The research conducted by [44] demonstrated that FA exhibited a protective effect against oxidative stress and photoaging. Also, Jung et al. [40] found that FA had hypoglycemic, hypolipidemic, and anti-inflammatory properties.

Furthermore, compared to the control group that did not receive any therapy, the group that received FA observed a decrease in the production of inflammatory markers, including IL-1β and TNFα. The anti-inflammatory impact of FA is explained by its ability to bind to AGEs and block their receptor (RAGE). This binding reduces pro-inflammatory cytokine production and prevents diabetic MI [18]. In line with the production of AGEs, it was shown that ISO-induced diabetic rats had a considerably higher expression of RAGE. However, this increase in RAGE expression was observed to be effectively blocked with the administration of FA.

According to earlier research, the delivery of an ISO injection causes a considerable rise in heart weight, heart rate, electrocardiographic changes, and cardiac marker enzyme levels (CK-MB, LDH), which provides strong evidence of the acute cardiotoxic effects of ISO injection [45]. According to the results of the present study, administering diabetic rats an ISO injection increased their heart weight, which in turn increased their heart weight-to-body weight ratio. There are several potential causes for the expansion of the heart’s intravascular space and weight, including increased protein and water contents [46]. The ISO-treated rats exhibited a higher level of various cytokines, namely TNF-α, IL-1β, and IL-6. It is known that cardiac toxicity is associated with higher levels of these cytokines [47]. TNF-α, IL-6, and IL-1β levels were significantly reduced in the rats that received FA treatment. Nevertheless, diabetic rats were significantly protected against the harmful effects of the ISO injection when pretreated with a greater dosage of FA.

Changes in the electrocardiogram (ECG) pattern have been identified as a dependable indicator of MI [9]. ECG abnormalities were observed in the group that received an ISO injection. These anomalies suggest an increase in the S.T. segment, a sign of myocardial ischemia [48]. Nevertheless, these alterations were reversed following the administration of FA in rats. This research shows that FA positively affects the electrical waveforms of rats’ hearts, which may indicate a protective effect.

The enzymes C.K., CK-MB, LDH, and CTnI are regarded as cardiac markers [49] and are therefore utilized as diagnostic markers for cellular injury. These enzymes most likely enter the circulation when myocytes sustain an injury that causes their cell membranes to burst or become more porous. Damaged myocytes are the main cause of these cardiac enzymes’ high levels [50]. Our investigation found that C.K., CK-MB, LDH, and CTnI levels were significantly elevated, but these cardiac marker enzyme levels were successfully restored after FA treatment. FA administered at a modest dose of 20 mg/kg exhibited a minimal inhibitory impact on cardiac marker enzyme levels; however, when administered at 40 mg/kg, FA revealed notable and major suppression. The release of enzymes that indicate cellular damage from cardiac cells may be prevented by returning biochemical parameters to their normal levels. Therefore, FA contributes significantly to the avoidance of metabolic changes in MI.

The histopathological analysis demonstrated that the administration of ISO in diabetic rats resulted in cellular structural and architectural damage, along with an escalation in the presence of neutrophils and swelling in the heart tissue, as previously documented by [51]. In contrast, administration of FA at the full dosage level led to decreased inflammatory lesions, accompanied by edema and a remarkable reduction in the extent of neutrophil infiltration. The research findings showed that the injection of FA effectively alleviated the harmful effects of ISO in diabetic rats. The results of this research provide credence to the idea that FA may protect the heart by lowering levels of oxidative stress. The restoration of normal biochemical indicators, the normalization of histopathological damage, and the reduction in oxidative stress reveal the powerful antioxidant effect of ferulic acid.

While the current study evaluated two concentrations of ferulic acid (20 mg/kg and 40 mg/kg), future studies should include a broader range of doses to establish a more comprehensive dose–response relationship. Also, future studies should investigate FA discontinuation before MI induction and its potential impact on the findings. This would help to identify the optimal therapeutic dose and further validate the dose-dependent cardioprotective effects of FA.

## 4. Materials and Methods

### 4.1. Experimental Animals

Forty male Wistar albino rats (75 days old), with an average weight of 150 ± 20 gm, were acquired from Vacsera, which specializes in biological goods and vaccinations (Vacsera, Egypt). The rats were kept in standard housing with unrestricted access to tap water and standard rat chow food. Other standard circumstances included regular light and dark cycles, a relative humidity of 55%, and a temperature of 25 ± 3 °C. Each group had eight animals. The Suez Canal University Faculty of Pharmacy’s Animal Care Committee approved all the experimental procedures (Ethical Number: 202205MA1).

### 4.2. Chemicals and Drugs

Streptozotocin (STZ) was obtained from Sigma-Aldrich (Munich, Germany) and freshly prepared using citrate buffer (0.1 M, pH = 4.5). Isoproterenol hydrochloride (ISO) was acquired from Sigma-Aldrich (Germany) and dissolved in a sterile saline solution. Ferulic acid (FA) was obtained from Career Henan Chemical Co., located in Zhengzhou, Henan Province, China. FA was suspended in a 0.2% carboxymethyl cellulose (CMC) aqueous solution. Ketamine hydrochloride was purchased from Egyptian International Pharmaceuticals Industries Company (EIPICO) (Cairo, Egypt), and xylazine hydrochloride was purchased from Adwia Pharmaceutical Company (Cairo, Egypt).

### 4.3. Induction of Type 2 Diabetes and Acute Myocardial Infarction

The rats were fed a diet high in fat for four weeks. After four weeks, the rats were fasted overnight and injected with freshly prepared streptozotocin at a dosage of 30 mg/kg, i.p. in a volume of 1 mL/kg [52]. Rats were fasted overnight (approximately 12 h) prior to STZ administration to ensure consistent fasting conditions across all animals. This fasting period aligns with the natural feeding cycle of rats, as they are nocturnal feeders and typically consume most of their food during the dark phase. Water was provided ad libitum during the fasting period to prevent dehydration. While the high-fat diet (HFD) was used to induce insulin resistance in the rats, glucose levels were not measured after the 4 weeks of HFD due to the focus of the study on the cardioprotective effects of ferulic acid in diabetic rats with isoproterenol-induced myocardial infarction. Diabetes was confirmed seven days after STZ administration by measuring fasting blood glucose levels, with levels exceeding 135 mg/dL considered indicative of diabetes. The blood glucose levels of each rat were measured using the One-Touch Ultra Mini glucometer seven days after STZ administration (USA). Rats with fasting blood glucose levels ≥ 135 mg/dL were considered diabetic, consistent with established protocols for STZ-induced type 2 diabetes models. This threshold reflects early-stage diabetes and aligns with clinical criteria for hyperglycemia in humans [5,9]. A dose of 50 mg/kg ISO was selected based on its ability to induce reproducible cardiac injury without excessive mortality. This dose is widely used in diabetic models to study the exacerbation of cardiac dysfunction due to heightened sympathetic activity [7,11]. The rationale behind this design was to evaluate the lasting effects of FA treatment after cessation, rather than its acute effects during the MI phase.

### 4.4. Experimental Design

For this investigation, 40 rats were randomly divided into five groups, with 8 in each group.

Group I (Control): Rats were given a regular, enjoyable diet. At the end of week 4, they were injected with citrate buffer (1 mL/kg, i.p.) as a control for STZ. In the tenth week, they received two doses of saline, the vehicle for isoproterenol.

Group II (Diabetic): Rats were administered a high-fat diet (HFD) for four weeks, after which they were given STZ at a specific dosage (30 mg/kg); then, rats were given two consecutive doses of saline, which served as the vehicle for isoproterenol at the tenth week.

Group III (Diabetic + MI): High-fat diet and STZ-induced diabetic rats were injected with two sequential subcutaneous dosages of isoproterenol (50 mg/kg/day) at the beginning of the tenth week.

Group IV (Diabetic + MI + low-dose FA): Rats received ferulic acid (20 mg/kg/day, P.O.).

Group V (Diabetic + MI + high-dose FA): Rats received ferulic acid (40 mg/kg/day, P.O.).

FA treatment (20 or 40 mg/kg/day) was initiated immediately after diabetes confirmation (week 4) and continued for 4 weeks until week 8. Isoproterenol (ISO, 50 mg/kg/day) was administered subcutaneously for two consecutive days at the beginning of week 10 to induce myocardial infarction (MI) [53].

### 4.5. Electrophysiology

Twenty-four hours after administering the second dosage of isoproterenol hydrochloride, the ECG was recorded from lead II for 2 min for each rat using the research Biopac Data Acquisition mp150 machine, ECG100 (BIOPAC system). The rats were administered thiopental sodium (20 mg/kg, I.P.) to induce anesthesia. Afterward, they were fastened to a board with adhesive tape to immobilize their legs, and the needle electrodes were inserted subcutaneously for the limb leads. The ground electrode was placed on the right leg. The negative (−ve) electrode was placed on the right arm. At the same time, the positive (+ve) electrode was placed on the left leg. The assessed ECG characteristics were heart rate (H.R.), R-R interval, S.T. segment amplitude, and T wave amplitude.

#### Blood Sampling

After the ECG recording, each rat had a 2 mL blood sample taken from its orbital vein for biochemical analysis. Immediate centrifugation at 2000× *g* for 15 min was performed on the blood samples following collection. The serum was chilled to −80 °C after separation until it was needed for various tests.

### 4.6. Processing of the Heart

At the termination of the research protocol, a surgical cut was performed in the abdomen, and the atria and great vessels were separated. Afterward, the hearts were extracted and washed using ice-cold phosphate-buffered saline. The hearts and left ventricles (LVs) were weighed to assess cardiac hypertrophy, and the ratio to body weight was estimated. Each ventricular myocardium was then separated into two distinct portions: The initial portion was reserved for the following biochemical assessments, while the second portion was subjected to overnight fixation at a concentration of 4% paraformaldehyde, followed by embedding in paraffin. The paraffin-embedded tissues were cut into sections with a thickness of 4 μm at the heart apex. These sections were then allowed to dry overnight at 37 °C. The sections were then treated to remove paraffin, rehydrated, and made ready for standard histological staining using hematoxylin and eosin (H&E).

### 4.7. Determination of Serum Cardiac Biomarkers

The measurement of blood cardiac enzymes was performed with pre-existing serum samples. The enzymatic kit developed by [54] was used to evaluate the activity of serum lactate dehydrogenase (LDH). The creatine phosphokinase (C.K.) activity was measured using a previously established technique [50]. An enzymatic approach was used to measure the activity of cardiac troponin I (cTnI) and serum creatine kinase-MB isoenzyme (CK-MB) [55]. An ultraviolet–visible spectrophotometer (UV-1601PC, Shimadzu, Tokyo, Japan) and commercial kits from Stanbio (Boerne, TX, USA) were used for all prior procedures.

### 4.8. Determination of TNF-α and MDA

After homogenizing, the cardiac samples were placed into the kits for the enzyme-linked immunosorbent assay (ELISA) for measuring malondialdehyde (MDA) and tumor necrosis factor-alpha (TNF-α). The assays were carried out using an automated ELISA reader (MyBioSource, San Diego, CA, USA) following the company’s specifications. The samples were measured twice, and the mean was used to represent them.

### 4.9. Histopathological Examination of Heart Tissues

A pathologist who was not informed of the therapy conducted a histopathological evaluation using a light microscope. The heart portions were analyzed as previously reported [56]. Grades 1, 2, 3, and 4 were assigned to the histological changes, signifying mild, moderate, high, and intensive pathological abnormalities, respectively.

The histopathological evaluation focused on identifying and grading the following pathological abnormalities: myocyte edema, mononuclear cell infiltration, necrotic myocytes, splitting of myofibers, areas of hemorrhage, loss of striations, and eosinophilic appearance of the sarcoplasm. These abnormalities were graded on a scale of 1 to 4, where Grade 1 signifies mild pathological changes, Grade 2 signifies moderate pathological changes, Grade 3 signifies high pathological changes, and Grade 4 signifies intensive pathological changes.

### 4.10. Determination of AGEs and RAGE by Quantitative Real-Time PCR Analysis Using Heart Tissue

The expression of advanced glycation end products (AGEs) and their receptor (RAGE) in heart tissue was quantified using a real-time polymerase chain reaction (PCR). Total RNA was extracted from heart tissue samples using a standard RNA isolation kit. cDNA synthesis was performed using reverse transcription, followed by amplification using the GoTaq^®^ 1-Step RT-qPCR System (https://worldwide.promega.com/products/pcr/qpcr-and-rt-qpcr/qpcr-kits/?tabset0=0&catNum=A6001 (accessed on 4 December 2024)). The following primer sequences were used for amplification:

- AGEs: Forward: GCT CTG ATA TCG GTG ACC CT; Reverse: TCG TAG TTG TGG TGG TCG AT.
- RAGE: Forward: GGA CAG TGT GGC TCG AAT CC; Reverse: CAA TTC CGA TAG CTG GAA GGA.
- β-actin (internal control): Forward: ACC CTA AGG CCA ACC GTG AA; Reverse: ACA GCC TGG ATG GCT ACG TAC A.

The PCR conditions included an initial reverse transcription step at 37 °C, followed by amplification cycles. The relative expression levels of AGEs and RAGE were normalized to β-actin and calculated using the 2^(−ΔΔCt) method. All reactions were performed in triplicate to ensure reproducibility.

#### Statistical Analysis

The data are presented as the mean values and the mean and standard deviation (S.D.). The results were evaluated using a one-way repeated measures analysis of variance (ANOVA), and pairwise comparisons were performed between all experimental groups using Bonferroni’s post hoc test to identify significant differences in key parameters, including body weight, glucose levels, cardiac enzymes, and inflammatory markers. The non-parametric data underwent analysis using the Kruskal–Wallis analysis of variance (ANOVA) method. The data were analyzed using the Statistical Package for the Social Sciences, version 25 (SPSS Software, SPSS Inc., Chicago, IL, USA). For mean differences, statistical significance was established at a significance level of *p* < 0.05.

## 5. Conclusions

This study demonstrates that ferulic acid (FA) exhibits promising cardioprotective effects in a diabetic rat model of isoproterenol-induced myocardial infarction (MI). The observed improvements in cardiac structure and function, the normalization of serum cardiac enzymes, and the reduction in blood glucose and insulin levels suggest that FA may play a beneficial role in mitigating diabetic cardiovascular complications. Additionally, FA treatment reduced the expression of pro-inflammatory cytokines and suppressed the upregulation of advanced glycation end products (AGEs) and their receptor (RAGE), indicating its potential to attenuate the inflammatory and oxidative stress pathways associated with diabetic MI.

However, it is important to note that these findings are based on a preclinical animal model, and further research is needed to confirm the efficacy and safety of FA in human studies. Future investigations should focus on elucidating the molecular mechanisms underlying FA’s cardioprotective effects and evaluating its long-term benefits in clinical settings. While our results are encouraging, they should be interpreted with caution, and FA should not yet be considered a definitive therapeutic option without further validation.

## Figures and Tables

**Table 1:** Impact of treatment with ferulic acid at doses of 20 or 40 mg/kg on body weight, left ventricular wet weight, and the ratio of heart weight to body weight (g/g) for different study groups.

Groups	Baseline BWt (g)	Final BWt (g)	% Change BWt	LV Weight to BWt (g/g) ×10^−3^	HtWt to BWt (g/g) ×10^−3^
Control	159.0 ± 3.6	187.7 ± 4.5 ^c^	18.0 ± 0.8 ^b^	1.1 ± 0.1 ^b^	3.7 ± 0.6 ^a^
Diabetic	159.0 ± 3.2	207.0 ± 3.7 ^b^	29.9 ± 4.3 ^ab^	1.3 ± 0.1 ^a^	4.1 ± 0.3 ^a^
Diabetic + MI	168.3 ± 2.1	226.0 ± 3.6 ^a^	34.3 ± 27 ^a^	1.3 ± 0.01 ^a^	4.3 ± 0.2 ^a^
Diabetic + MI + FA (20 mg/kg)	166.3 ± 2.7	202.7 ± 5.4 ^bc^	22.0 ± 5.6 ^b^	1.2 ± 0.1 ^ab^	4.1 ± 0.2 ^a^
Diabetic + MI + FA (40 mg/kg)	163.1 ± 4.4	191.7 ± 3.7 ^bc^	17.9 ± 4.7 ^b^	1.2 ± 0.1 ^ab^	4.0 ± 0.1 ^a^
*F* test	2.48	11.39	6.54	5.07	1.41
*p*-Value 0.05	0.111ns	0.0010 **	0.0075 **	0.017 **	0.29

** and different superscript letters indicate significant differences at *p* < 0.05.

**Table 2:** The impact of administering ferulic acid at 20 or 40 mg/kg on fasting glucose levels, insulin concentration (unit/L), and insulin resistance in several groups was examined (n = 8 per group).

Groups	BGL (mM/L)	Insulin (μIU/mL)	HOMA-IR Index
Control	5.13 ± 0.06 ^b^	4.90 ± 0.26 ^e^	1.77 ± 0.06 ^c^
Diabetic	7.90 ± 0.52 ^b^	17.20 ± 0.20 ^c^	5.70 ± 0.44 ^bc^
Diabetic + MI	10.60 ± 2.04 ^a^	37.20 ± 2.65 ^a^	17.60 ± 3.99 ^a^
Diabetic + MI + FA (20 mg/kg)	6.1 ± 0.10 ^b^	20.60 ± 2.65 ^b^	6.43 ± 0.91 ^b^
Diabetic + MI + FA (40 mg/kg)	6.00 ± 0.26 ^b^	9.47 ± 0.95 ^d^	2.97 ± 0.60 ^bc^
*F* test	6.32	155.49	34.16
*p*-Value 0.05	0.008 **	<0.0001 **	<0.0001 **

** and different superscript letters indicate significant differences at *p* < 0.05.

## Data Availability

The original contributions presented in this study are included in the article. Further inquiries can be directed to the corresponding author.
